# Advancing Personalized Medicine Through FDM 3D Printing: Ketoprofen Tablets with Customizable Drug Release Profiles and In Silico Simulation

**DOI:** 10.3390/pharmaceutics17111495

**Published:** 2025-11-19

**Authors:** Haya Khader Ahmad Yasin, Moawia M. Al-Tabakha, Siok Yee Chan

**Affiliations:** 1 School of Pharmaceutical Sciences, Universiti Sains Malaysia, Pulau Pinang 11800, Malaysia; haya.yasin@gmail.com; 2 Department of Pharmaceutical Sciences, College of Pharmacy and Health Sciences, Ajman University, Ajman P.O. Box 346, United Arab Emirates; 3 Centre of Medical and Bio-Allied Health Sciences Research, Ajman University, Ajman P.O. Box 346, United Arab Emirates

**Keywords:** 3D printing, fused deposition modeling, hot-melt extrusion, ketoprofen, filaments, immediate-release, sustained-release, personalized medicine, GastroPlus, IVIVC

## Abstract

**Background/Objectives**: Fused deposition modeling (FDM) three-dimensional (3D) printing represents an emerging manufacturing platform for personalized oral dosage forms. Its success relies on developing robust drug-loaded filaments with consistent mechanical, thermal, and dissolution properties. This work aims to (i) develop and characterize ketoprofen-loaded filaments using hot-melt extrusion (HME) and (ii) utilize them to fabricate both immediate-release (IR) and sustained-release (SR) tablets via FDM 3D printing. **Methods**: Filaments were prepared using Kollicoat^®^ IR and hydroxypropyl methylcellulose (HPMC, 2600–5600 cP) as functional polymers. Sorbitol and sodium lauryl sulfate (SLS) were incorporated as plasticizer and surfactant, respectively. Filaments were evaluated for quality attributes, drug content, tensile strength, and physicochemical and surface characteristics using Scanning Electron Microscopy (SEM), Attenuated Total Reflection Fourier-transform infrared (ATR-FTIR), X-ray Diffraction (XRD), Differential Scanning Calorimetry (DSC) and Thermogravimetric Analysis (TGA). Optimized filaments were fed into an FDM 3D printer to fabricate ketoprofen tablets with varied geometries, shell numbers, and infill densities. Tablets were subjected to USP tests (weight variation, friability, hardness, disintegration, assay, content uniformity), dissolution profiling, and release kinetics modeling. Comparative dissolution studies with market Profenid^®^ and Bi-Profenid^®^ tablets were conducted. GastroPlus^®^ simulations were used for in vitro–in silico correlation. **Results:** Among the tested formulations, Kollicoat^®^ IR-based filaments with sorbitol and SLS (F6) demonstrated superior printability, characterized by consistent feeding, stable extrusion, and reliable formation of uniform structures for immediate-release applications. In contrast, HPMC-based filaments with sorbitol (F13) offered the most robust performance for SR formulations. Both exhibited uniform diameter, drug loading, and mechanical strength. IR tablets achieved >80% release within 30 min, while SR tablets prolonged release up to 12 h, following Higuchi and Korsmeyer–Peppas kinetics. All quality attributes complied with USP limits. Market products showed comparable dissolution, validating the approach. GastroPlus^®^ simulations predicted pharmacokinetic profiles consistent with reported data, supporting IVIVC. **Conclusions:** This integrated workflow establishes a robust strategy for producing IR and SR ketoprofen tablets from a single FDM platform. The results highlight the feasibility of point-of-care, personalized medicine using 3D printing technologies.

## 1. Introduction

Additive manufacturing (AM), particularly fused deposition modeling (FDM), is increasingly recognized as an innovative approach for pharmaceutical manufacturing and personalized drug delivery. By enabling precise control over dosage form geometry and internal architecture, FDM allows the design of tailored drug release profiles and individualized therapy matrices [1,2].

Unlike conventional manufacturing methods, which often require separate processing steps for different release profiles, FDM integrates digital design and material science, offering on-demand customization, cost-efficiency, and patient-centered solutions [3,4].

The success of pharmaceutical FDM hinges on producing robust, drug-loaded filaments, typically via hot-melt extrusion (HME). These filaments must combine adequate mechanical strength, thermal stability, and homogeneous drug distribution to ensure consistent printability and therapeutic performance [5,6].

Challenges in filament fabrication include optimizing polymer selection, ensuring adequate drug–excipient miscibility, and controlling extrusion parameters to mitigate issues such as brittleness, thermal degradation, and nozzle clogging during printing [7,8].

Ketoprofen, a non-steroidal anti-inflammatory drug (NSAID), is an ideal model compound due to its clinical relevance and physicochemical properties. It exhibits moderate solubility and a crystalline nature, making it suitable for evaluating drug–polymer interactions, thermal processing, and dissolution behavior under 3D printing (3DP) conditions. Ketoprofen is clinically used in both immediate-release (IR) formulations for acute pain and sustained-release (SR) formulations for chronic therapy profiles [9]. Developing both dosage types on a single FDM platform presents a significant step toward adaptable, personalized therapy.

Previous studies have reported FDM-printed ketoprofen tablets, typically focusing on IR or SR, but rarely combining both within the same workflow [10,11,12]. Moreover, many have investigated limited design parameters, leaving gaps in understanding the interplay between formulation, digital design, and drug release. A distinctive aspect of this study is integrating a comprehensive quality control assessment, which facilitates systematic evaluation of the reproducibility, uniformity, and robustness of 3D-printed ketoprofen dosage forms together with their release profiles. The study addresses the gaps by integrating two phases: (i) development and characterization of ketoprofen-loaded filaments using HME, and (ii) fabrication and evaluation of 3Dp ketoprofen tablets with IR and SR profiles. Comparative studies with market tablets and in silico simulations using GastroPlus^®^ provided translational insights.

## 2. Materials an Methods

### 2.1. Materials

Ketoprofen (≥98% purity) was obtained from Sigma-Aldrich (Merck KGaA, Darmstadt, Germany). Polymers included Kollicoat^®^ IR (polyvinyl alcohol–polyethylene glycol graft copolymer, purity ≥ 98%), hydroxypropyl methylcellulose (HPMC, viscosity 2600–5600 cP, purity ≥ 99%), polyvinyl alcohol (PVA) (purity ≥ 99%), ethyl cellulose (purity ≥ 98%), Kollidon^®^ SR (purity ≥ 98%), methyl cellulose (purity ≥ 99%), polyvinylpyrrolidone (PVP) (purity ≥ 99%) and hydroxypropyl cellulose (HPC) (purity ≥ 99%) obtained from Sigma-Aldrich (Merck KGaA, Darmstadt, Germany). Excipients included sodium lauryl sulfate (SLS), Tween 80, Span 20, and sorbitol (Loba Chemie Pvt. Ltd., Mumbai, India). Organic solvents (ethanol, methanol, acetonitrile ≥ 99.8% purity) were from Merck (Darmstadt, Germany). Market reference tablets Profenid^®^ 100 mg and Bi-Profenid^®^ 150 mg (Sanofi, Paris, France) were purchased from a local pharmacy for comparison.

### 2.2. Methods

#### 2.2.1. Filament Fabrication via HME

HME was performed using Noztek Pro Touch extruders (Noztek Ltd., Shoreham-by-Sea, UK). Extrusion parameters (temperature: 120–180 °C; screw speed: 10–50 rpm) were optimized based on thermal analysis of ketoprofen and polymers. Three drug loadings (10, 30, 50% *w*/*w*) were tested. Sorbitol and surfactants were incorporated as plasticizers/wettability enhancers. Blends were prepared via (i) geometric mixing, (ii) melt incorporation, or (iii) solvent-assisted blending. The extrudates were collected directly from the hot-melt extruder, cooled to room temperature to form continuous filaments, and stored in a desiccator until further use.

#### 2.2.2. Filament Images

The images of filaments were taken with a high-resolution digital camera on a cell phone.

#### 2.2.3. Filament Scanning Electron Microscopy (SEM)

Surface morphology of selected IR and SR tablets fabricated filaments were examined using a Hitachi S-4800 SEM (JEOL JSM-7600F). Samples were fixed on aluminum stubs with carbon tape, sputter-coated with gold for 10 s, and imaged at 30 kV under varying magnifications.

#### 2.2.4. Filament Quality Control

The quality of the extruded filaments was assessed through weight variation, diameter uniformity, and drug content uniformity in accordance with pharmacopeial guidelines. Ten 5 cm samples from each filament batch were weighed using a digital balance (Shimadzu, Kyoto, Japan) with weighing boats for weight variation. Each sample’s mean weight and percentage deviation were calculated according to the USP Uniformity of Dosage Units.

Ten filament segments (5 cm each) were selected for diameter measurement. The diameter of each segment was measured at three evenly spaced points along the length (beginning, midpoint, and end) using a digital caliper (Insize, Guangzhou, China). The average diameter and standard deviation were determined.

Drug content uniformity was assessed by weighing and crushing ten samples of 0.4 g filament each. The powdered samples were dissolved in 2000 mL of methanol to achieve a concentration of 25 µg/mL, sonicated for one h, filtered, diluted, and analyzed by UV spectrophotometry (Shimadzu, Kyoto, Japan). The expected and actual drug content were compared, and the results were expressed as mean ± standard deviation (SD) [13].

#### 2.2.5. Filament Tensile Strength

Tensile testing was performed to evaluate the mechanical integrity of the extruded filaments using a Shimadzu Autograph AGS-X universal testing machine (Shimadzu Corporation, Kyoto, Japan) with a 500 N load cell and Trapezium X software version 1.5.4. Filaments (60 mm length) were conditioned at 25 ± 2 °C and 50 ± 5% RH for 24 h, then mounted vertically with a gauge length of 30 mm. Tests were conducted at a crosshead speed of 5 mm/min following a modified ASTM D882 method for filaments [14]. Parameters were recorded, including break force, break stress, elongation, strain, displacement, maximum force, and break time [14,15]. Each formulation was tested in triplicate (*n* = 3), and results were reported as mean ± SD.

#### 2.2.6. Filament Dissolution Studies

Drug release from filaments was evaluated using a USP-II apparatus (UDT-812, LOGAN, Shanghai, China) with an autosampler and controllers. A two-phase dissolution method was applied due to the pH-dependent solubility of ketoprofen. For the first 120 min, 750 mL of 0.1 M HCl (pH 1.2) was used, followed by the addition of 250 mL trisodium phosphate to adjust pH to 7.4, simulating intestinal conditions (120 min for IR, 600 min for SR). Tests were performed at 37 °C with a paddle speed of 50 rpm. Samples (3 mL) were withdrawn at predetermined intervals, filtered (0.45 μm), and analyzed at 260 nm using a Shimadzu spectrophotometer (Tokyo, Japan). All experiments were conducted in triplicate, expressing the results as mean ± SD.

#### 2.2.7. Tablet Design and Dimensions

**Figure 1:**
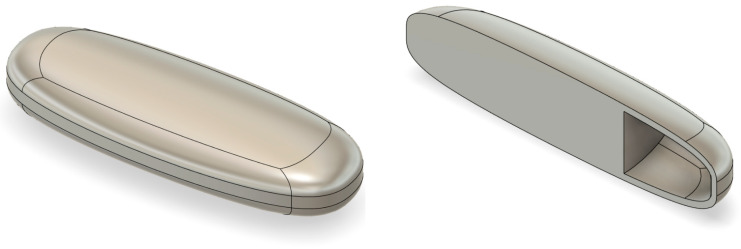
Design of the 3DP elongated tablet using Autodesk Fusion 360: external view of the elongated tablet (**left**); cross-sectional view showing the internal air chamber (**right**).

**Figure 2:**
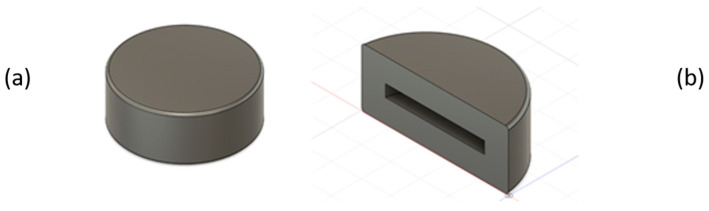
Design of the 3DP round tablet using Autodesk Fusion 360: (**a**) external view of the round tablet; (**b**) cross-sectional view showing the internal air chamber.

Elongated and round tablets were designed using Autodesk Fusion 360^®^ (Autodesk Inc., San Rafael, CA, USA) and exported as STL files, as shown in Figure 1 and Figure 2. Two designs were created: one with an air compartment and one without. SR tablets shared the same design but differed in size and printing patterns. Printing parameters (nozzle temperature, shell number, and infill density) were systematically varied.

#### 2.2.8. Selection of Dose

Ketoprofen was selected as the API for this study. It has poor solubility in aqueous media (0.01 mg/mL at 25 °C) and is classified as a BCS Class II drug. Due to its physicochemical properties, ketoprofen is a well-studied model drug in various formulations. Various doses were evaluated, specifically 75 mg, 100 mg, and 150 mg.

#### 2.2.9. Images

Images of 3DP tablets were taken with a high-resolution digital camera of a cellphone.

#### 2.2.10. Tablet Quality Control

Tablet evaluation included weight variation, dimensions, hardness, disintegration, friability, assay, and content uniformity. Ten tablets per formulation were individually weighed (Shimadzu balance, Kyoto, Japan), and the mean weight with percentage deviation was calculated per USP. Diameter and thickness were measured with a digital caliper (Insize, Guangzhou, China), and hardness was assessed on ten samples using a YD-1 tester (LABOAO, Zhengzhou, China). Disintegration testing followed USP <701> with six tablets placed in a disintegration apparatus (Erweka, Haan, Germany) at 37 ± 2 °C in 1000 mL distilled water, and results were statistically compared by one-way ANOVA. Friability was tested with ten tablets with an Erweka TA 100 friabilator at 25 rpm for 4 min (100 rotations), with <1% considered acceptable. Ten tablets were tested for content uniformity, which was determined spectrophotometrically at 260 nm after individual tablet dissolution, sonication, dilution, and filtration. For assay, twenty powdered tablets equivalent to 200 mg ketoprofen were analyzed by HPLC (Shimadzu, Japan), with results required within 90–110% of label claim.

#### 2.2.11. Tablet In Vitro Studies

Drug release from 3DP tablets was evaluated using a USP-II apparatus (UDT-812, LOGAN, China) with autosampler and system controllers. Owing to the pH-dependent solubility of ketoprofen, a two-phase method was applied: 750 mL of 0.1 M HCl (pH 1.2) for the first 120 min to simulate gastric conditions, followed by the addition of 250 mL trisodium phosphate to adjust the pH to 7.4, representing intestinal fluid for 120 min (IR) or 600 min (SR). The paddle speed was set at 50 rpm, and the temperature was maintained at 37 °C. Samples (3 mL) were withdrawn at predetermined intervals, filtered through 0.45 µm membranes, and analyzed spectrophotometrically at 260 nm using a Shimadzu spectrophotometer (Tokyo, Japan) with a 1 mm flow-through cuvette. Samples with absorbance values exceeding 1.2 were diluted with dissolution medium to remain within the validated linear range of the calibration curve. All measurements were performed in triplicate, and results are reported as mean ± SD.

#### 2.2.12. Comparison with Market Tablets

Profenid^®^ 100 mg and Bi-Profenid^®^ 150 mg tablets (Sanofi, Paris, France) were analyzed for assay and dissolution to benchmark the 3DP formulations. Tests were conducted under the same conditions as described for the 3DP tablets to ensure comparability.

#### 2.2.13. Tablet Micro-CT Scan

The internal structure, density, and porosity of the tablets were analyzed using a Scanco Micro-CT 100 (SCANCO Medical AG, Brüttisellen, Switzerland) at 90 kV and 200 µA with a voxel size of 10 µm, producing 1700–2300 slices per sample. The acquisition time was approximately 60 min, and image reconstruction with 3D rendering was performed using Scanco Evaluation Software V6.6.

#### 2.2.14. Tablet Attenuated Total Reflection Fourier-Transform Infrared (ATR-FTIR) Spectroscopy

ATR-FTIR spectra were obtained using an IRAffinity-1S spectrometer (Shimadzu, Japan) over 400–4000 cm^−1^, averaging 50 scans at 4 cm^−1^ resolution. Pure ketoprofen, HPMC, Kollicoat^®^ IR, their mixtures, filaments, and 3DP tablets were analyzed to investigate possible drug–polymer interactions following hot-melt extrusion and FDM printing.

#### 2.2.15. Tablet Scanning Electron Microscopy (SEM)

Surface morphology of selected IR and SR tablets was examined using a Hitachi S-4800 SEM (JEOL JSM-7600F). Samples were fixed on aluminum stubs with carbon tape, sputter-coated with gold for 10 s, and imaged at 30 kV under varying magnifications.

#### 2.2.16. Tablet XRD (X-RAY Diffractometry)

Crystalline and amorphous characteristics of ketoprofen, polymers, physical mixtures, and tablets were determined using an X-ray diffractometer (Rigaku, Tokyo, Japan). Data were collected from 15° to 70° 2θ at a scanning speed of 0.005° s^−1^ using Cu-Kα radiation (λ = 1.542 Å) in grazing-incidence mode at 1°.

#### 2.2.17. Tablet Thermogravimetric Analysis (TGA)

Thermal stability of pure components, selected filaments, and tablets (10 mg) was evaluated using a LABSYS evo TGA (Setaram, Caluire-et-Cuire, France) from 30 to 500 °C at 10 °C/min under argon flow (40 mL/min). Weight changes were recorded using a microbalance with ±0.1 mg precision.

#### 2.2.18. Tablet Differential Scanning Calorimetry (DSC)

DSC analysis was performed on pure components, selected filaments, and tablets using LABSYS evo DSC (Setaram, Caluire-et-Cuire, France). Approximately 10 mg of each sample was heated to 250 °C at 10 °C/min under argon (40 mL/min) in sealed aluminum pans. Two heating cycles were conducted to assess melting transitions and glass transition temperatures.

#### 2.2.19. In Silico Simulation

GastroPlus^®^ v10.1 was used to simulate plasma concentration–time profiles using the ACAT model. Inputs included solubility, log P, pKa, and permeability from literature. IR and SR tablet release profiles were imported. Predicted pharmacokinetics (Cmax, Tmax, AUC) were compared with clinical data.

#### 2.2.20. Statistical Analysis

SPSS v26 was used. Data are expressed as mean ± SD. ANOVA with Tukey’s post hoc test assessed differences in release and quality parameters. Regression models explored correlations between design parameters and drug release. The significance level was *p* < 0.05.

## 3. Results and Discussion

### 3.1. Filament Fabrication via HME

Three distinct techniques were employed to prepare feedstock materials for HME, each tailored to address different formulation challenges and optimize processability.

The first and most straightforward approach involved geometric mixing of all dry components, including ketoprofen, the selected polymer, and excipients using a mortar and pestle. This conventional method ensured uniform API distribution across the powder blend. After achieving a consistent mix, the powders were directly introduced into the extruder [4,10]. To enhance powder flow during feeding, slight downward pressure was applied with the pestle, helping guide the material into the screw.

The second technique involved first melting the polymer, then gradually incorporating the active pharmaceutical ingredient and other excipients into the molten matrix. The resulting mixture was allowed to solidify at room temperature before being manually chopped into small, uniform pieces suitable for extrusion [16].

The third method utilized solvent-assisted blending. Ketoprofen and the polymer were dissolved and homogenized in ethanol using a magnetic stirrer for 30 min. The solution was then spread onto a tray and left to partially dry. Once semi-solid, the material was cut into small fragments and further dried at 70 °C for six hours to ensure complete solvent evaporation [17,18,19]. All prepared filaments were stored in a vacuum desiccator at room temperature before printing. Table 1 summarizes the composition and processing parameters of the drug-loaded filaments fabricated via hot-melt extrusion. This table provides a detailed overview of the polymers and additives used, along with key extrusion parameters such as temperature, screw speed, and drug content, which were systematically varied to optimize filament quality and printability.

All polymers and excipients used in this study Kollicoat^®^ IR, HPMC, sorbitol, and SLS are pharmaceutically approved and listed in the FDA Inactive Ingredients Database as “Generally Recognized as Safe” [20] for oral use. Their established safety and compatibility with common formulation processes support the translational feasibility of this 3D printing approach. The increasing regulatory recognition of additive manufacturing, as evidenced by the FDA approval of Spritam^®^, further underscores the potential of FDM-printed oral dosage forms for future clinical application [21].

### 3.2. Filament Images

**Figure 3:**
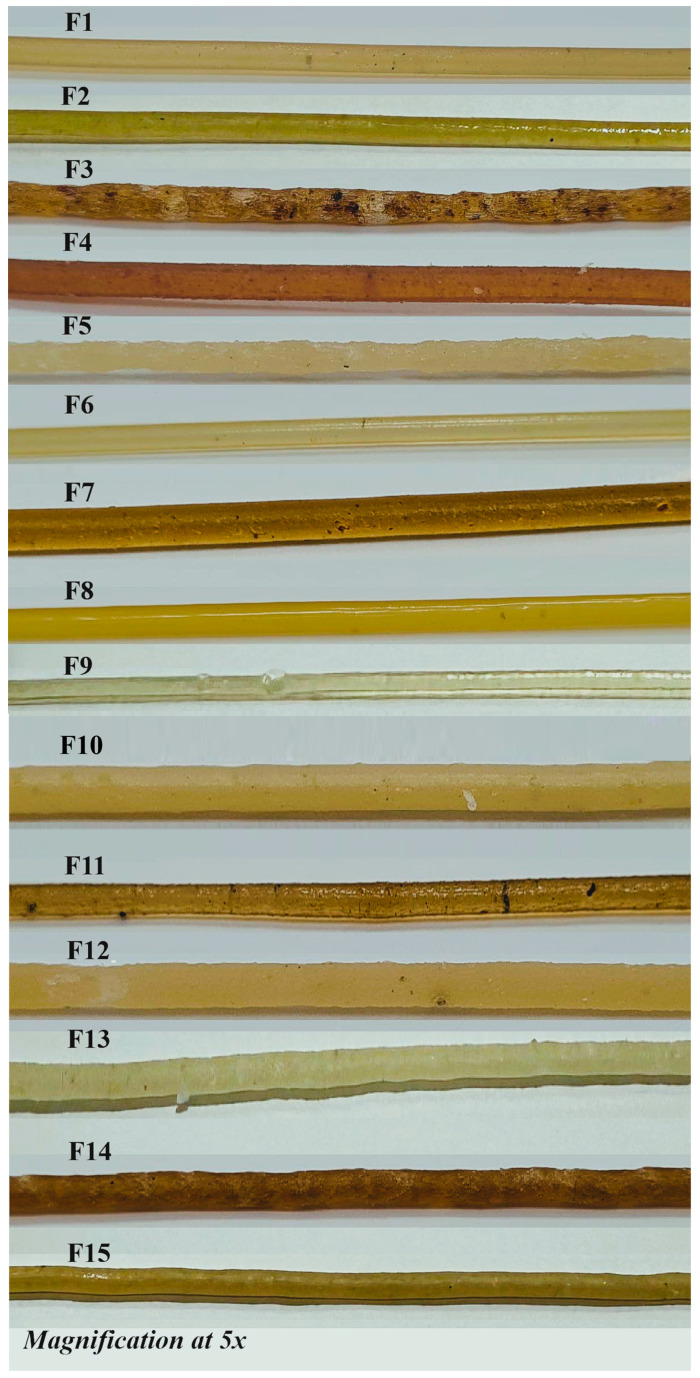
Photographs of extruded filaments F1–F15 fabricated by hot-melt extrusion. Color and surface uniformity varied with drug load, processing temperature, and excipient addition, with optimized F6 and F13 demonstrating smooth surfaces and minimal defects suitable for FDM 3D printing.

Filaments were successfully fabricated using HME, initially exhibiting a pale-yellow color (Figure 3). A concentration-dependent darkening was observed with increasing ketoprofen loading, progressing from pale yellow (F1) to brownish discoloration at 50% drug content (F3), where filaments appeared burnt and morphologically inconsistent, including irregular geometry and surface defects. This was attributed to the lower melting point of ketoprofen (94 °C) compared to Kollicoat^®^ IR (200 °C). Elevated temperature (180 °C, F4) further intensified discoloration, while higher screw speed (50 rpm, F5) produced rough, poorly blended filaments due to reduced residence time. Incorporating excipients also influenced filament quality: sorbitol, SLS, and Tween increased darkening, with sorbitol likely undergoing caramelization at processing temperatures. Nevertheless, sorbitol improved filament plasticity, yielding smoother and more uniform filaments (F6) than rough and brittle filaments lacking sorbitol (F1). This likely reflects partial caramelization of sorbitol and increased drug–polymer interaction at elevated concentrations, consistent with the amorphization observed in XRD and DSC results in Section 3.16 and Section 3.18, respectively. Overall, drug concentration, extrusion conditions, and excipient composition significantly affected filament color, morphology, and quality.

### 3.3. Filament SEM

**Figure 4:**
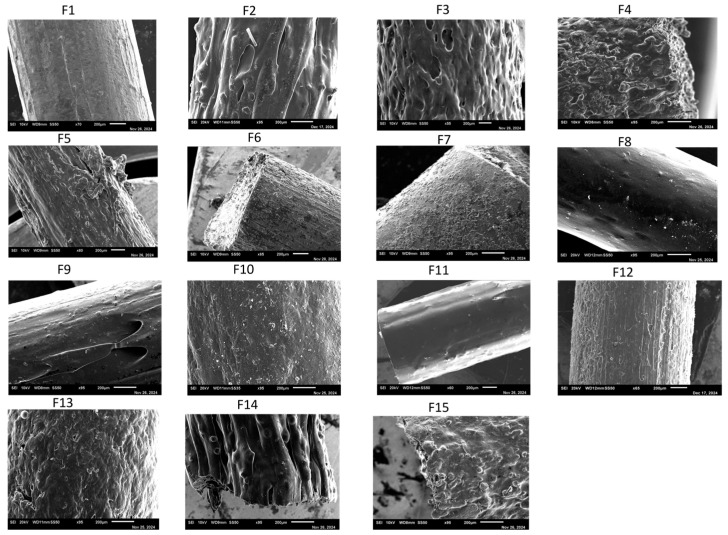
SEM images of filaments F1–F15 showing the effect of polymer type, drug loading, and additives on microstructure. Low drug loads produced smooth, compact surfaces, whereas higher loads induced crystallinity and porosity. Sorbitol- and surfactant-containing filaments (e.g., F6, F13) exhibited dense, homogeneous morphologies favorable for amorphous drug dispersion.

SEM of filaments F1–F15, shown in Figure 4, revealed that polymer type, drug loading, extrusion parameters, and the inclusion of additives strongly influenced surface texture, internal compactness, and drug dispersion. Kollicoat^®^ IR-based filaments (F1–F8) were generally cylindrical and continuous. Low drug load filaments (F1, 10%) exhibited smooth, compact surfaces. In contrast, higher loadings (F2–F3, 30–50%) showed increasing surface roughness, porosity, and crystalline domains, indicative of phase separation or recrystallization when the polymer’s solubilizing capacity was exceeded. Elevated extrusion temperature (F4, 180 °C) improved filament uniformity, while high screw speeds (F5, 50 rpm) induced surface roughening and crystallinity due to reduced residence time.

Additives improved filament morphology: sorbitol and surfactants (F6–F8, F13–F15) promoted dense, smooth, and layered structures with minimal crystalline features, enhancing drug-polymer miscibility and amorphous drug dispersion. PVA-based filaments (F9) exhibited smooth, dense surfaces without crystallinity, highlighting excellent solubilizing properties. In contrast, less hydrophilic polymers like Kollidon^®^ SR (F10) and ethyl cellulose (F11) displayed rough surfaces and pronounced crystalline domains due to poor miscibility with ketoprofen. HPMC-based filaments (F12–F15) were more compact and homogeneous, particularly with additive incorporation, confirming the role of hydrophilic excipients in reducing crystallinity.

Overall, SEM analysis demonstrated that high drug load promotes crystalline ketoprofen formation, while hydrophilic polymers and additives maintain an amorphous, molecularly dispersed state, enhancing solubility and potential dissolution. These findings emphasize the critical influence of polymer selection, drug load, processing conditions, and additive use on filament microstructure and drug distribution [22,23].

### 3.4. Filament Quality Control

The 15 extruded filaments (F1–F15) demonstrated relatively consistent mean diameters (1.550–1.819 mm) and weights (0.097–0.162 g), with low variability (SDs ≤ 0.021 mm for diameter and ≤0.0042 g for weight). The actual drug content ranged from 77.11 ± 4.01% (F11) to 102.11 ± 2.15% (F8), reflecting the effects of formulation composition, drug loading, and processing parameters. Filaments F1–F9, designed for IR, generally showed slightly lower to moderate drug content (82–102%), whereas SR filaments F10–F15 maintained higher uniformity in drug loading (89–99%), indicating controlled incorporation of ketoprofen within polymer matrices. The physical characteristics of the extruded filaments, including mean weight, diameter, and actual drug content, are summarized in Table 2.

### 3.5. Filament Tensile Strength

**Figure 5:**
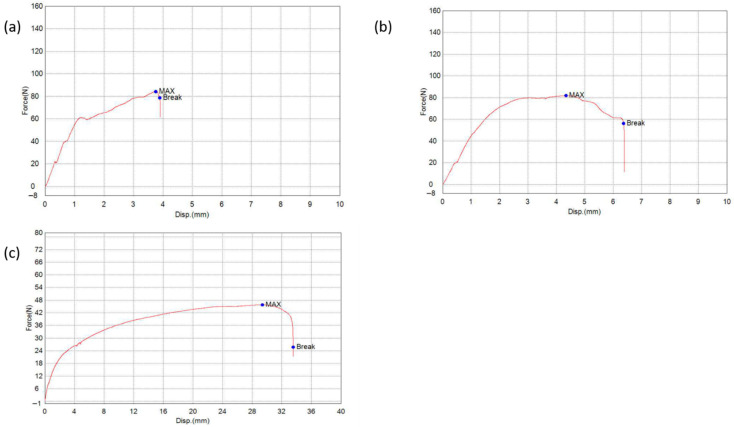
Tensile force–displacement curves for (**a**) F6, (**b**) F13, and (**c**) reference PVA MakerBot filament (drug-free). The red line represents the tensile profile obtained during the stretching test, showing the relationship between applied force and displacement until failure. The blue dots indicate key mechanical points on the curve: MAX, corresponding to the maximum tensile force before yielding, and Break, representing the point of filament fracture.

The mechanical performance of the fabricated filaments was strongly influenced by extrusion parameters, particularly screw speed (rpm) and temperature, as well as the incorporation of additives such as Sorbitol, SLS, and Tween is presented in Table 3. Moderate screw speeds (e.g., 30 rpm, F2) enhanced break force and extensibility due to improved melt mixing and polymer fusion, whereas excessively high speeds (F5) led to weaker, less cohesive filaments, likely from reduced residence time and incomplete polymer homogenization. Within the tested range (160–180 °C), extrusion temperature had minimal effect on Kollicoat^®^ IR filaments, indicating a broad thermal processing window, Figure 5 shows an example of filament F6 and F13 compared to reference PVA.

The addition of sorbitol, a small-molecule plasticizer, markedly increased filament strength and resilience by enhancing polymer chain mobility, improving interfacial bonding, and producing a denser filament structure (e.g., F6 vs. F2). Improved tensile strength with sorbitol facilitated filament handling and ensured uninterrupted extrusion during FDM. Mechanically robust filaments translate to more uniform tablets, directly influencing dosing reproducibility. Incorporation of SLS alongside Sorbitol further improved mechanical stability, likely by enhancing dispersion and reducing interfacial tension between drug and polymer phases. In contrast, Tween, a non-ionic surfactant, increased filament flexibility and elongation (e.g., F7, F15) but reduced tensile strength, indicating greater ductility at the expense of mechanical robustness.

Overall, these results demonstrate that optimal filament properties are achieved through careful balancing of extrusion parameters and additive composition, in line with previous reports in the FDM and hot-melt extrusion literature, where intermediate screw speeds and plasticizer inclusion maximize mechanical performance while maintaining printability and structural integrity [24].

### 3.6. Filament Dissolution

**Figure 6:**
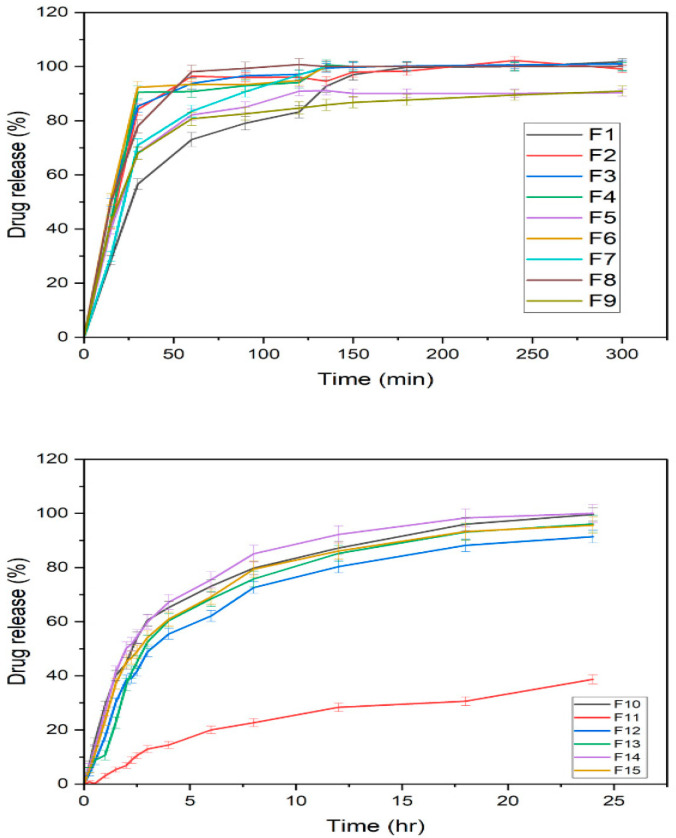
Dissolution studies of filament formulations (F1–F15). (mean ± SD, *n* = 6).

Dissolution rate profiles of IR and SR filaments are shown in Figure 6. IR filaments (F1–F9) exhibited rapid ketoprofen release, primarily influenced by polymer type, drug loading, processing temperature, and excipient inclusion. Increasing drug loading in Kollicoat^®^ IR-based filaments (F1–F3) enhanced dissolution rates, with F3 achieving nearly complete release within 2 h. The faster release in F2 compared with F3 suggests that drug dispersion within the polymer matrix is critical; higher loading reduces wettability and slows dissolution. Higher extrusion temperatures (F4) slightly improved dissolution, whereas higher mixing speed (F5) marginally slowed it. The inclusion of solubilizers (sorbitol and surfactants, F6–F8) further accelerated release, with F6 achieving complete dissolution within 2.5 h. Due to inherent hydrophilicity and matrix erosion, PVA-based filament F9 also showed rapid release (>100% in 4 h). SR filaments (F10–F15) exhibited prolonged drug release, as expected, attributable to the use of hydrophobic or rate-controlling polymers. This demonstrates the successful modulation of ketoprofen release profiles via polymer selection and filament design.

### 3.7. Selection of Filaments

Following hot-melt extrusion trials, two filaments, F6 (Kollicoat^®^ IR-based) and F13 (HPMC-based), were selected for 3D printing of ketoprofen tablets due to their superior printability and mechanical performance. F6 exhibited smooth extrusion, minimal brittleness, consistent flow, and uniform diameter, making it suitable for IR tablets with well-defined geometry. F13 demonstrated sufficient flexibility and mechanical strength for SR applications, maintaining structural integrity and producing robust matrix-type tablets with uniform infill and excellent layer adhesion despite the absence of surfactants.

Both filaments were extruded under optimized temperature and screw speed conditions to achieve homogeneous drug–polymer dispersion, then manually fed into the FDM printer without spooling or drying, consistent with contemporary pharmaceutical 3D printing practices. The successful printability of F6 and F13 confirmed their mechanical suitability and processing compatibility with FDM, and their selection aligned with the desired release profiles, IR for F6 and SR for F13, establishing reliable platforms for on-demand ketoprofen tablet fabrication.

### 3.8. Design Dimensions and Printing Parameters of 3D-Printed Tablets

The 3D-printed (3DP) ketoprofen tablets were fabricated with varying drug doses, release profiles, and internal designs to evaluate the impact of geometry and printing parameters on printing efficiency and tablet characteristics.

**Figure 7:**
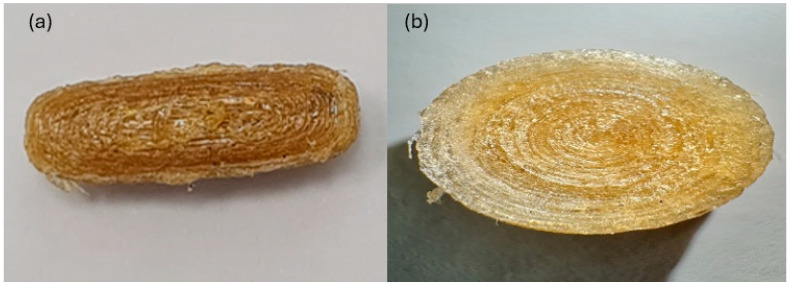
Photographic images of a 3DP (**a**) elongated tablet, (**b**) Round tablet top view.

Visual inspection, as shown in Figure 7, revealed that the disk-shaped tablets possessed a more symmetric and uniform shape in some prints. The continuity of curvature and absence of sudden edges facilitated uniform filament deposition, resulting in a less rugged surface texture. The long tablets exhibited a less symmetrical surface with ridges and non-uniform layering. These were attributed to their greater surface area and more than one edge, which appeared to support filament accumulation during the printing process.

Due to their improved surface quality and reproducibility, the round tablet shape was used for further investigations. Although elongated tablets are theoretically more suited for IR preparations, the round tablets were preferred due to their superior visual and physical characteristics [25].

Table 4 summarizes the body dimensions and average printing times for IR and SR tablets, showing that larger sizes and SR designs required longer printing durations. Table 5 details the specific formulation characteristics and printing parameters for all tested tablets (T1–T21), including shell number, infill density, printing pattern, speed, extrusion temperature, dimensional accuracy, drug content, and polymer type. These data provide a comprehensive overview of the design and process variables optimized to achieve reproducible, mechanically robust, and accurately dosed 3DP tablets.

### 3.9. Tablet Images

**Figure 8:**
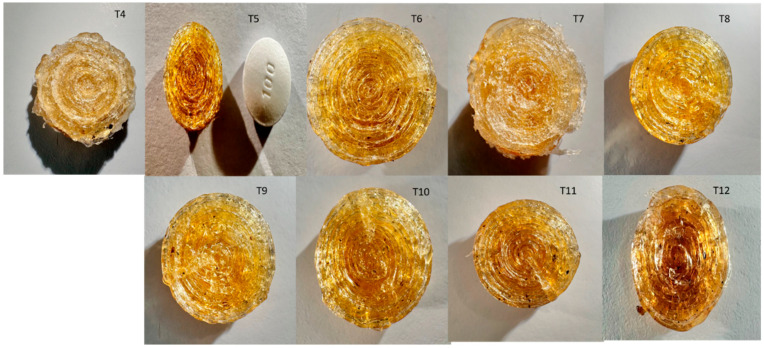
Top-view images of 3DP round tablets (Formulations T4–T12) fabricated using FDM with varying process parameters and compositions. The images illustrate the characteristic concentric deposition layers and surface morphology differences among the tested formulations. For reference, a market immediate-release tablet, Profenid^®^ 100 mg (Sanofi-Aventis, France), is shown alongside formulation T5 for size and appearance comparison.

The physical appearance, dimensional uniformity, and printability of 3D-printed ketoprofen tablets were strongly influenced by printing parameters, including infill density, shell number, printing temperature, and infill pattern, as illustrated in Figure 8. Tablets with only 1% infill (T1–T3) were structurally fragile and failed upon removal from the print bed, even with increased shell numbers, demonstrating that very low infill is insufficient for maintaining tablet integrity.

Tablets printed with 25–50% infill (T4–T12) successfully retained their cylindrical shape. One-shell tablets (e.g., T4, T7) exhibited surface irregularities due to insufficient shell support, whereas increasing the shell number to 5–10 (T5, T6, T8, T9) improved geometry, surface smoothness, and dimensional consistency. Among these, T5 (25% infill, 5 shells) provided the optimal balance of shape retention, surface quality, and uniformity, closely matching the size and thickness of the market Profenid^®^ 100 mg tablet, although it maintained a layered FDM structure rather than a compressed, coated appearance.

Printing temperature affected tablet coloration: most tablets printed at 180 °C showed the characteristic amber-yellow of Kollicoat^®^ IR, while higher temperatures (200 °C, T12) caused darker discoloration, suggesting potential thermal degradation. Alternative infill patterns, such as tri-hexa (T10) and grid (T11), produced denser, more uniform internal geometries, potentially enhancing mechanical strength and controlled drug release.

**Figure 9:**
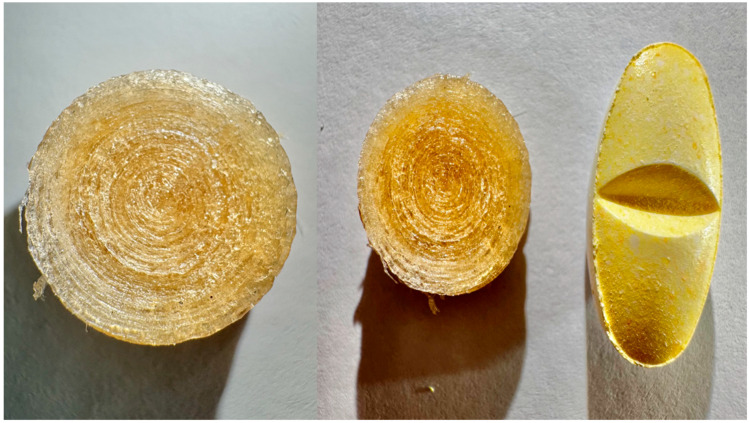
Top-view images of 3DP round tablet (Formulation T20) produced by FDM, shown alongside a market sustained-release reference tablet, Bi-Profenid^®^ 150 mg (Sanofi-Aventis, France).

Overall, successful 3D-printed tablets required a minimum infill density of 25% and at least five shells to achieve reproducible, well-formed dosage units. T5 demonstrated optimal macroscopic and printing characteristics, while T20 (SR formulation) also produced visually robust tablets comparable to the market Bi-Profenid^®^ 150 mg, highlighting the capability of FDM to produce patient-ready oral dosage forms, as shown in Figure 9.

### 3.10. Tablet Quality Control

The physical characterization of the selected 3DP ketoprofen tablets (T5, T13, T14, T20, and T21) demonstrated consistent and reproducible quality across all formulations, as summarized in Table 6. Tablet weights ranged from 0.170 ± 0.004 g (T13, IR 50 mg) to 0.537 ± 0.008 g (T21, SR Air 150 mg), with all units meeting pharmacopeial limits for weight uniformity. Diameters and thickness closely matched the designed specifications, with minor variations attributed to layer-by-layer deposition and thermal expansion during FDM printing. IR tablets showed disintegration times within the USP-acceptable range, while SR formulations (including air-pocket designs) did not disintegrate, confirming their prolonged-release functionality. Hardness values were higher in SR tablets due to increased shell numbers and infill density, whereas friability remained below 1% for all formulations, indicating sufficient mechanical robustness. Assay and content uniformity results confirmed precise and homogeneous drug loading across the different designs, with relative standard deviations below 2%. Overall, these findings highlight the capability of FDM 3D printing to produce tablets with reproducible dimensions, mechanical strength, and accurate drug content, even with complex geometries incorporating internal air compartments.

### 3.11. Tablet In Vitro Dissolution

**Figure 10:**
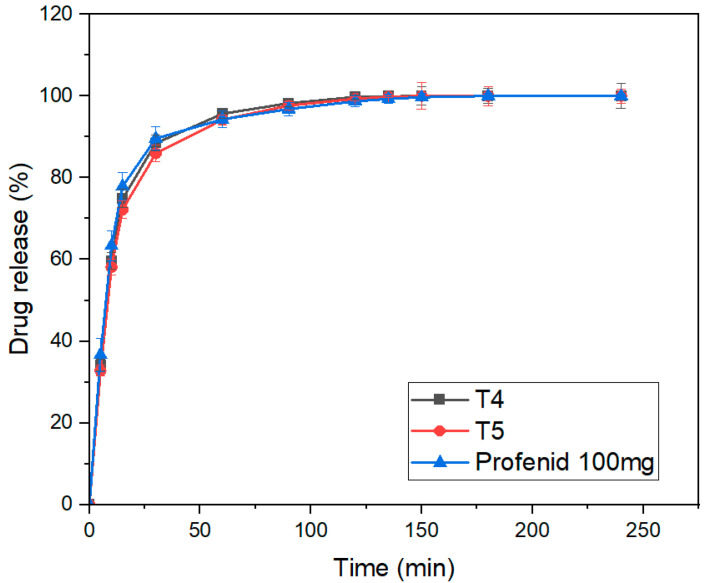
In vitro dissolution profile of T4–T5 and market Profenid^®^ 100 mg (mean ± SD, *n* = 6).

The dissolution profiles of the 3DP IR tablets T4 and T5 were compared with Profenid^®^ 100 mg using a USP apparatus over 240 min (*n* = 6), as illustrated in Figure 10. Both released >80% of drug within 30 min, confirming IR behavior. T4 showed faster and more consistent dissolution, reaching 75% at 15 min versus slightly less for T5, reflecting a minor delay due to its higher shell number. After 30 min, all formulations converged to near-complete release (100%) with low variability. Rapid release from T5 reflects increased surface wettability and reduced diffusion path length, aligning with the intended IR function. Overall, both were effective IR formulations, with T4 most closely matching the reference.

**Figure 11:**
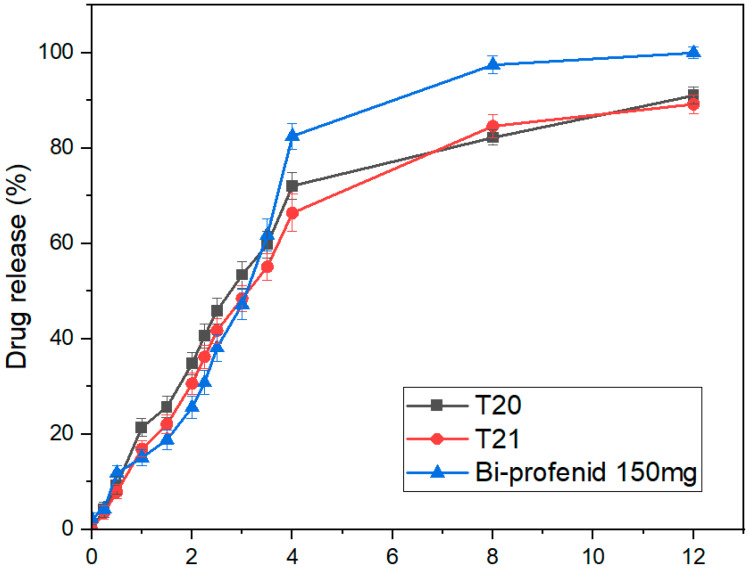
In vitro dissolution profile of T20–T21 and market Bi-Profenid^®^ 150 mg (mean ± SD, *n* = 6).

In contrast, the dense HPMC matrix in T20 slowed medium penetration, sustaining release through polymer swelling and erosion. The SR profiles of T20 and T21 were compared with Bi-Profenid^®^ 150 mg over 12 h, as illustrated in Figure 11. Both showed gradual, extended release, slower than the reference (which reached >80% by 6 h and almost 100% by 8 h). T21, incorporating an internal air compartment for buoyancy, exhibited a slight initial lag (up to 4 h) before aligning with T20 by 6 h. Both maintained consistent release with low variability (*n* = 6). These findings confirm SR over 12 h, with T21’s buoyant design modestly modulating early dissolution without affecting total release, supporting its potential as a gastro-retentive strategy. The delayed dissolution observed in T21 (air-compartment design) relative to T20 underscores the influence of tablet architecture on release kinetics. The hollow internal chamber reduced the overall density and effective surface area exposed to dissolution medium during the initial hours, slowing water penetration into the matrix. This resulted in a prolonged wetting phase and delayed drug diffusion. Once hydration progressed and the surrounding shell eroded, dissolution accelerated and converged with the solid-core T20 profile. This lag effect highlights how geometric modifications, not just polymer choice, can fine-tune release kinetics. Such gastro-retentive floating structures could be advantageous in prolonging gastric residence time, but they also introduce variability in early drug release, which must be considered in translational applications.

### 3.12. Comparison with Market Tablets

The dissolution profiles of T4 and T5 closely matched the reference Profenid^®^ 100 mg, as confirmed by model-independent metrics in Table 7. T4 showed excellent similarity with f_1_ = 1.59% and f_2_ = 78.4, indicating minimal early-stage differences and strong overall profile alignment. T5 also met regulatory criteria with f_1_ = 2.87% and f_2_ = 74.6, reflecting slightly higher variability likely due to formulation or processing differences. Both formulations comply with EMA and FDA standards (f_1_ < 15%, f_2_ > 50), with T4 providing a closer replication of the reference, especially during initial dissolution [26].

For SR tablets, T20 closely resembled Bi-Profenid^®^ 150 mg, with f_1_ = 8.93% and f_2_ = 62.4, indicating minimal absolute differences and strong overall similarity as listed in Table 8. T21, featuring an internal air compartment, exhibited slightly higher early-phase deviation (f_1_ = 12.6%) but maintained f_2_ = 56.2, meeting regulatory thresholds. These results demonstrate that T20 best replicates the reference release profile, while T21 achieves modified release through design variations without compromising overall dissolution over 24 h. Similarity factors (f2 > 50) for T5 vs. Profenid^®^ suggest bioequivalence at the dissolution stage, underscoring the feasibility of replacing conventional products with 3D-printed alternatives. For SR tablets, partial similarity reflects design-driven modulation of release, opening opportunities for tailoring therapy beyond market formulations.

### 3.13. Micro-CT

Micro-CT was employed to non-destructively evaluate the internal structure, porosity, and material density of 3DP tablets fabricated via FDM. Three visualizations were generated for each tablet: 3D surface-rendered models for overall shape and printing accuracy, cross-sectional images to assess infill patterns and shell thickness, and binarized images to quantify solid versus void regions. The main quantitative metric, BV (Bone Value) material mean density, indicated the compactness of each formulation and correlated with mechanical strength and dissolution behavior.

IR tablets with lower shell numbers and infill (T4: 1 shell, 25% infill, BV = 0.6286) exhibited highly porous, discontinuous internal structures, supporting rapid disintegration. Increasing shell count (T5: 5 shells, 25% infill, BV = 0.6297) improved peripheral reinforcement but retained internal porosity. Intermediate designs with higher infill (T8: 5 shells, 50% infill, BV = 0.6357; T9: 10 shells, 50% infill, BV = 0.636) displayed denser cores and fewer voids, balancing faster onset with moderate release. Higher infill density was observed in T8 with reduced pore channels, correlating with its slower dissolution relative to T5. SEM images confirmed surface porosity, providing a mechanistic basis for water ingress and drug diffusion patterns.

SR configurations with maximal shells and infill (T20: 100 shells, 100% infill, BV = 0.6521) showed thick, continuous shells and homogeneous matrices, favoring slower drug release. Incorporation of an internal air compartment (T21: 100 shells, 100% infill, BV = 0.6374) reduced overall density but successfully reproduced the designed hollow space, potentially enabling delayed erosion or gastro-retentive effects.

**Figure 12:**
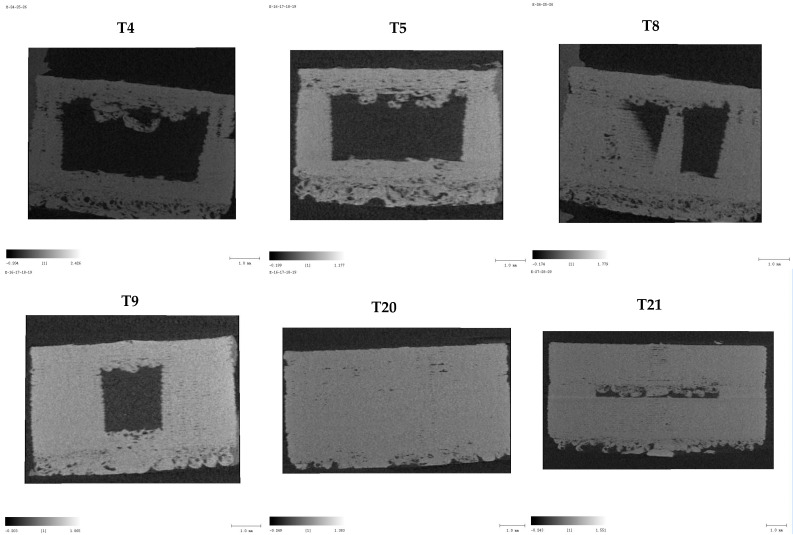
Micro-CT cross-sectional images of 3D-printed ketoprofen tablets (T4, T5, T8, T9, T20, and T21) showing the influence of infill density, shell number, and internal air compartments on internal structure and porosity.

Overall, as illustrated in Figure 12, Micro-CT confirmed that design parameters, including shell number, infill percentage, and internal compartments, were accurately translated into the physical tablets, directly influencing porosity, density, and predicted release profiles.

**Figure 13:**
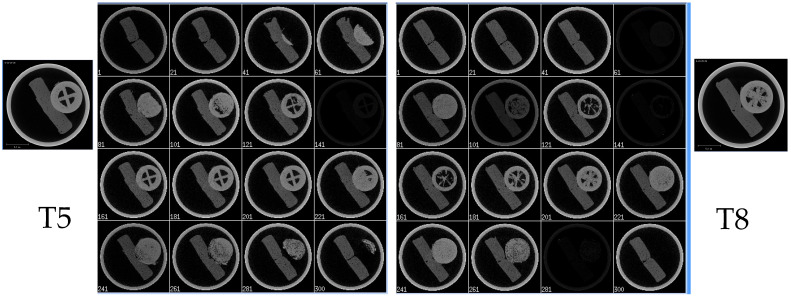
Axial CT slice for T5 and T8.

Axial CT slices Figure 13 further highlighted the effect of infill density: T5 (25% infill) displayed fragmented internal matrices with extensive air-filled regions, whereas T8 (50% infill) showed higher greyscale intensity, continuous filament paths, and markedly reduced porosity. These images confirm that digital design parameters, such as shell number, infill percentage, and internal compartments, are accurately translated into the final printed tablets, influencing structural integrity and predicted dissolution profiles.

### 3.14. Attenuated Total Reflection Fourier-Transform Infrared (ATR-FTIR) Spectroscopy

**Figure 14:**
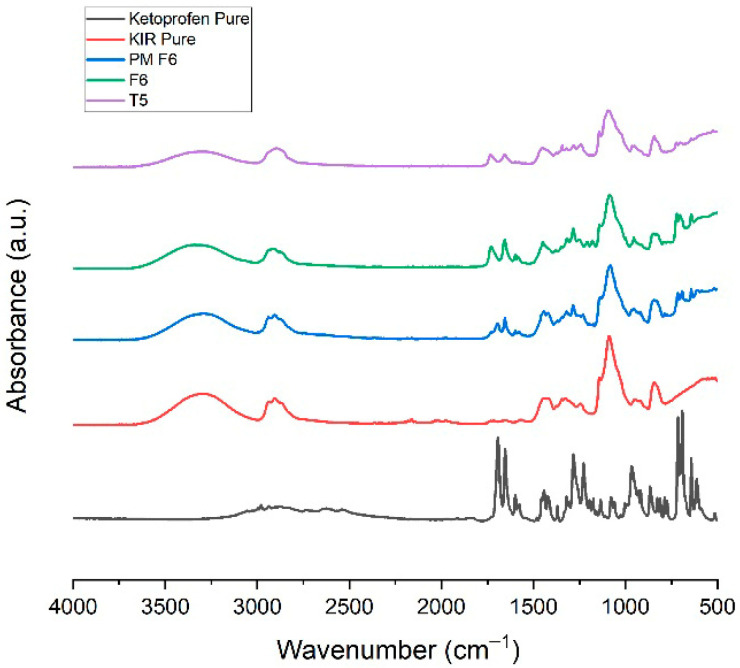
ATR-FTIR spectra for Ketoprofen, KIR, PM, F6, and T5.

The ATR-FTIR spectra of ketoprofen, Kollicoat^®^ IR (KIR), their physical mixture (PM F6), extruded filament (F6), and 3DP tablet (T5), as illustrated in Figure 14, showed progressive changes indicating increasing drug–polymer interactions. Characteristic ketoprofen peaks at 1697 and 1651 cm^−1^ (carbonyl stretches) were retained in the physical mixture, suggesting no chemical interaction, but were attenuated in the filament and further in the printed tablet. Broadening of the O–H region indicated hydrogen bonding between ketoprofen’s carboxylic group and KIR’s hydroxyl groups, while the overall reduction in peak intensity suggested partial molecular dispersion or amorphization during FDM printing. No new characteristic peaks were observed, suggesting no major chemical modifications during extrusion or printing [27].

**Figure 15:**
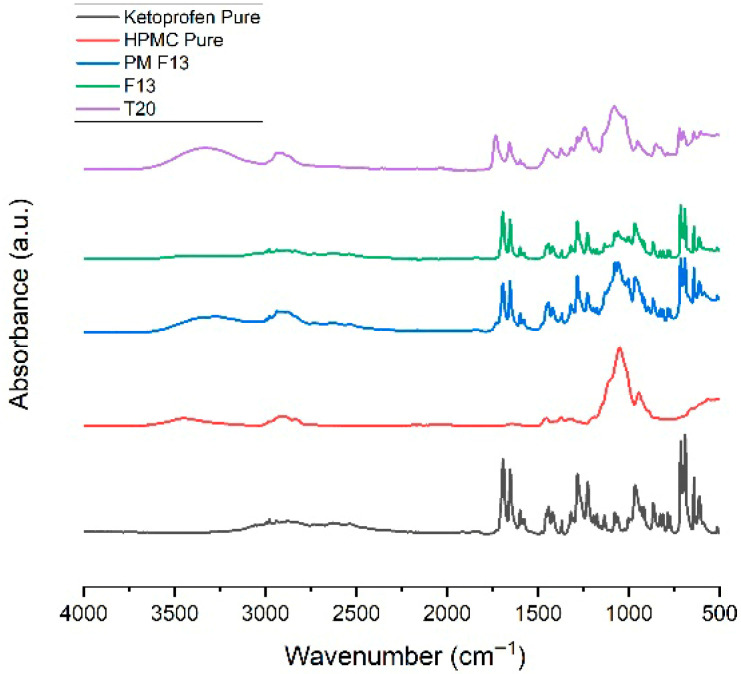
ATR-FTIR spectra for Ketoprofen, HPMC, PM, F13, and T20.

As shown in Figure 15, the ATR-FTIR spectra of ketoprofen, HPMC, their physical mixture (PM SR6), extruded filament (F13), and 3DP tablet (T20) revealed similar trends. The physical mixture preserved all characteristic peaks, indicating no interaction prior to processing. After extrusion and printing, the ketoprofen carbonyl and aromatic signals were progressively attenuated, with broadening of the hydroxyl bands, consistent with hydrogen bonding and enhanced molecular dispersion into the HPMC matrix. These spectral changes suggest partial amorphization and increased miscibility of ketoprofen within the polymer, supporting the SR performance while confirming the drug’s chemical stability [27].

### 3.15. Scanning Electron Microscopy (SEM)

**Figure 16:**
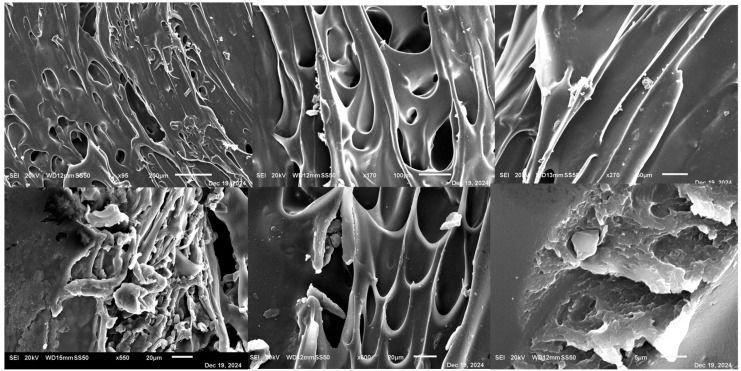
SEM images of immediate-release tablet T5 at varying magnifications: top row (left to right) 95×, 170×, 270×; bottom row (left to right) 550×, 600×, and 2700×. The images reveal a porous microstructure with microvoids and incomplete filament fusion, consistent with rapid disintegration and fast drug release.

SEM images in Figure 16 of T5 revealed a porous, web-like surface with numerous microvoids and thin polymer shells, reflecting the low infill (25%) and moderate shell count [5] used in its FDM printing. Filament fusion appeared incomplete at intermediate magnifications, but layer adhesion remained sufficient to preserve structural integrity. Higher magnifications showed embedded ketoprofen particles, crack-like channels, and fragmented polymer strands, creating a matrix conducive to rapid water ingress, erosion, and fast drug release. Overall, the microstructure supports IR behavior while maintaining adequate mechanical stability for handling.

**Figure 17:**
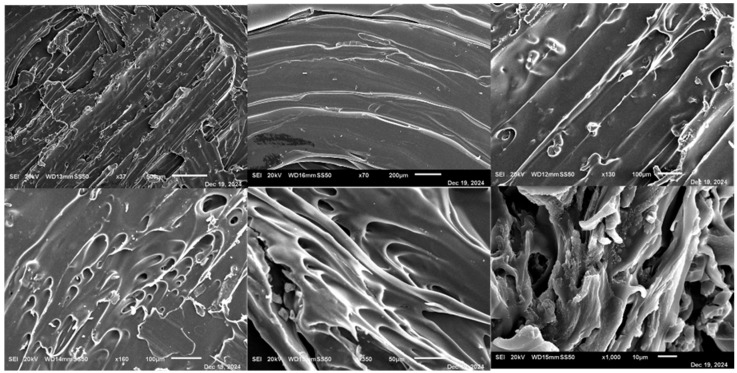
SEM images of sustained-release tablet T20 at varying magnifications: top row (left to right) 37×, 70×, 130×; bottom row (left to right) 160×, 350×, and 1000×. The images reveal a continuous lamellar structures and amorphous drug dispersion within the HPMC matrix, supporting prolonged drug release.

In contrast, as shown in Figure 17, T20 exhibited a well-organized lamellar structure with parallel, continuous layers and strong interlayer adhesion, reflecting optimized FDM parameters and the use of HPMC filaments. Higher magnification images showed a fused, amorphous polymer matrix embedding the drug, with minimal microporosity and no crystalline ketoprofen visible, indicating substantial amorphization or molecular dispersion. This cohesive architecture provides mechanical robustness, controlled disintegration, and sustained drug release, highlighting the effectiveness of hot-melt extrusion combined with FDM for fabricating uniform, high-resolution SR tablets [24].

### 3.16. XRD

**Figure 18:**
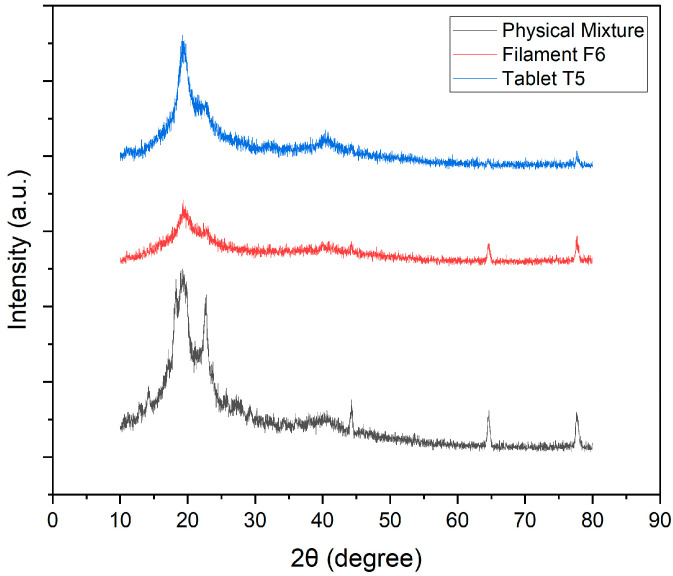
XRD patterns of ketoprofen–Kollicoat^®^ IR physical mixture, F6 fabricated filament, and T5 3D-printed tablet.

**Figure 19:**
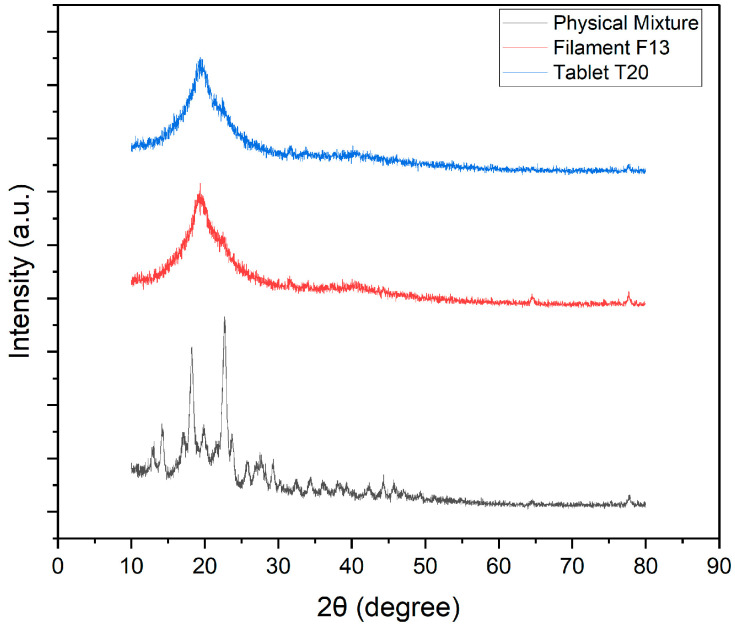
XRD patterns of ketoprofen–HPMC physical mixture, F13 fabricated filament, and T20 3D-printed tablet.

XRD analysis was conducted to examine the crystalline properties of ketoprofen and assess its structural modifications during processing into filaments and subsequent 3DP tablets T5 and T20. The diffractograms of the physical mixture (a), extruded filament (b), and 3DP tablet (c) are presented in Figure 18 and Figure 19. The physical mixture showed sharp, intense peaks between 10° and 30° 2θ, characteristic of crystalline ketoprofen. After hot-melt extrusion, the filament exhibited reduced peak intensity and a broad halo around 20°, indicating partial amorphization. In the final 3DP tablets (T5 and T20), the peaks were further diminished, with a broad halo dominating the diffractogram, confirming that ketoprofen was largely amorphous. This transformation reflects successful dispersion of the drug within the polymer matrices during extrusion and FDM printing, consistent with enhanced solubility and modified release profiles reported in previous studies [1]. The inclusion of sorbitol played a dual role in filament development. Mechanistically, sorbitol acts as a small-molecule plasticizer, reducing intermolecular hydrogen bonding among polymer chains and thereby lowering the glass transition temperature (Tg). This enhanced chain mobility during hot-melt extrusion facilitated molecular dispersion of ketoprofen within the polymer matrix, explaining the disappearance of crystalline peaks in XRD and DSC. At the same time, sorbitol’s hydrophilic nature promoted drug–polymer miscibility and water uptake, which further supported amorphization. The caramelization and surface darkening observed at higher processing temperatures likely reflected partial thermal degradation of sorbitol; however, this effect was outweighed by its contribution to filament smoothness and mechanical robustness, as evidenced by higher tensile strength and reduced brittleness. Thus, sorbitol was not simply a plasticizer but a key stabilizer of the amorphous state, directly impacting dissolution performance. The absence of crystalline peaks in XRD and DSC indicates amorphization of ketoprofen, which is expected to enhance solubility and dissolution rate. This structural transition validates the role of HME in producing solid dispersions suitable for FDM.

### 3.17. Thermogravimetric Analysis (TGA)

**Figure 20:**
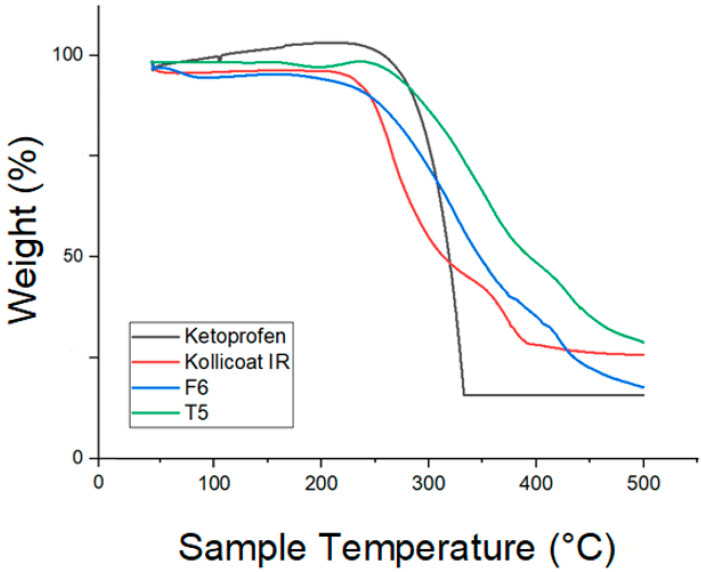
TGA results for Ketoprofen, KIR, F6 and T5.

TGA of pure Ketoprofen showed a sharp weight loss between 280 and 320 °C, reflecting its thermal decomposition, while Kollicoat^®^ IR exhibited two-step degradation, with initial moisture loss near 100 °C and major polymer breakdown from 270 to 450 °C. The extruded filament F6 displayed an earlier onset of weight loss (~240 °C) and a smoother, more gradual degradation profile, suggesting drug–polymer interactions and partial amorphization during hot-melt extrusion. The 3DP tablet T5 showed an even broader degradation range (230–500 °C), indicating combined degradation of the polymer and drug, and confirming uniform dispersion and structural reorganization of ketoprofen within the Kollicoat^®^ IR matrix. These results, illustrated in Figure 20, support FDM as a thermally viable process for IR formulations [28,29]. 

**Figure 21:**
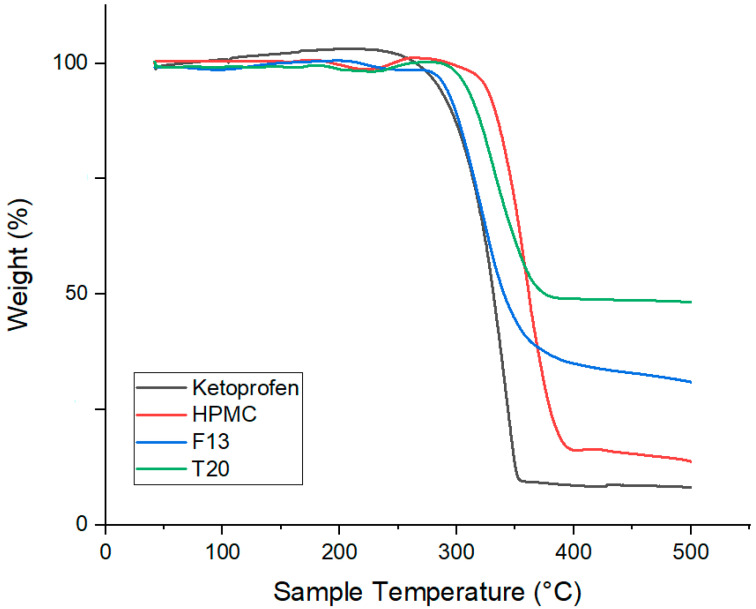
TGA results for Ketoprofen, HPMC, F13, and T20.

Pure Ketoprofen again exhibited a sharp single-step decomposition starting around 280 °C, while HPMC displayed minor moisture loss near 100 °C and major polymer degradation between 300 and 400 °C. The extruded filament F13 showed intermediate thermal behavior, with weight loss beginning at 250 °C and extending to 430 °C, indicating effective dispersion and drug–polymer interactions without compromising stability. The 3DP tablet T20 demonstrated a broad decomposition range from 240 to 450 °C, reflecting uniform drug distribution and robust thermal integrity after printing. The TGA results illustrated in Figure 21 confirm that both HME (160 °C) and FDM printing (180 °C) do not compromise the thermal stability of Ketoprofen or HPMC, validating the process for SR oral dosage forms [28,29].

### 3.18. Differential Scanning Calorimetry (DSC)

**Figure 22:**
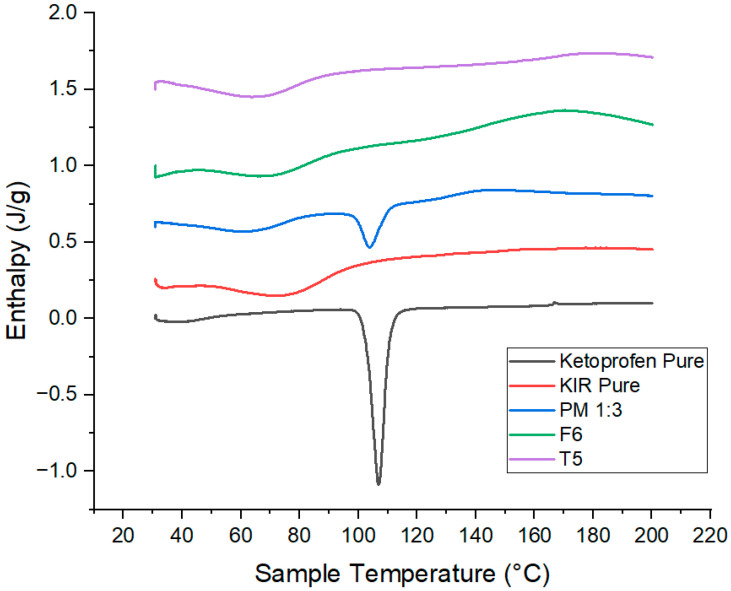
DSC thermograms for Ketoprofen, KIR, PM, F6 and T5.

The thermal behavior of ketoprofen, KIR, F6, and T5 was evaluated using DSC, and Figure 22 illustrates the results. DSC of pure ketoprofen displayed a sharp endothermic peak at ~96 °C, confirming its crystalline nature, while Kollicoat^®^ IR showed no melting transitions, indicating an amorphous polymer. The physical mixture exhibited a reduced and broadened ketoprofen peak, suggesting minor interactions and partial crystallinity loss. The melting peak in the extruded filament F6 disappeared entirely, indicating successful amorphization and molecular dispersion within the polymer. This amorphous state was preserved in the 3DP tablet T5, confirming that FDM processing maintains drug amorphicity and supports potential improvement in solubility and bioavailability [28,29].

**Figure 23:**
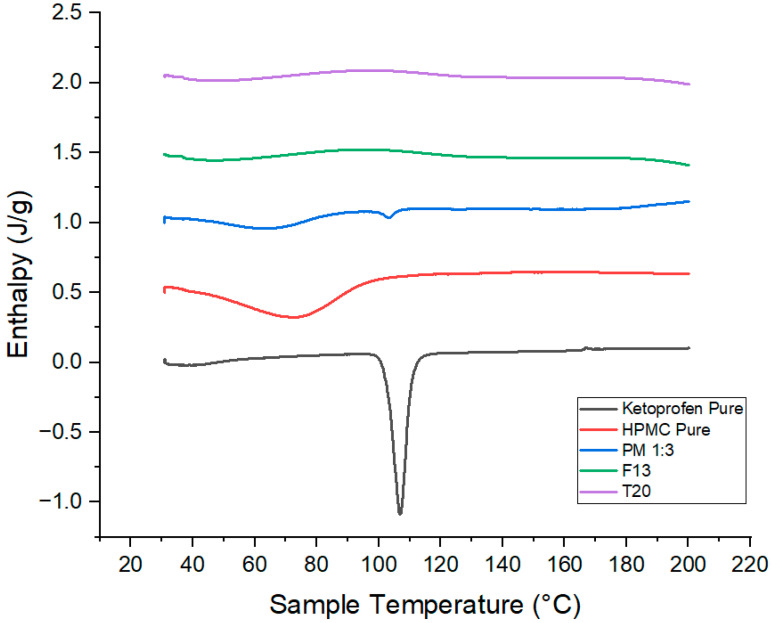
DSC thermograms for ketoprofen, HPMC, PM, F13 and T20.

Pure ketoprofen again showed a sharp melting peak at ~96 °C, while HPMC displayed no endotherms, consistent with its amorphous nature. The physical mixture showed a diminished and broadened ketoprofen peak, indicating partial crystallinity reduction. The extruded filament F13 exhibited complete disappearance of the drug’s melting peak, confirming amorphization and uniform dispersion in HPMC. The 3DP tablet T20 maintained this amorphous state after printing, demonstrating that hot-melt extrusion combined with FDM preserves drug amorphicity, facilitating stable solid dispersions with potential for enhanced dissolution and bioavailability [4]. Figure 23 represents the results of DSC thermograms.

### 3.19. In Silico Simulation

**Figure 24:**
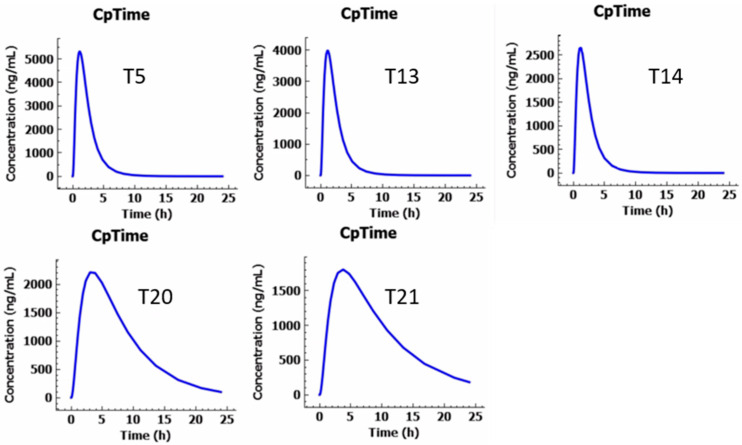
Simulated plasma concentration–time profiles generated using GastroPlus^®^ for T5 (100 mg), T13 (75 mg), T14 (50 mg), T20 (150 mg), T21 (150 mg).

GastroPlus^®^ simulations accurately predicted the pharmacokinetic behavior of ketoprofen formulations, highlighting clear differences between IR and SR designs as listed in Table 9. IR tablets (50–100 mg) exhibited rapid absorption, with Tmax around 1 h and dose-proportional Cmax ranging from 2500 to 5400 ng/mL. Plasma concentrations declined quickly, reflecting ketoprofen’s short half-life and rapid elimination, and total exposure (AUC) increased linearly with dose, consistent with literature reports. Regional absorption predominantly occurred in the duodenum and jejunum, with minimal gastric uptake. Simulated plasma concentration–time profiles generated using GastroPlus^®^ for T5 (100 mg), T13 (75 mg), T14 (50 mg), T20 (150 mg), and T21 (150 mg) are shown in Figure 24. SR formulations (150 mg) demonstrated prolonged absorption and lower peak plasma concentrations. The standard SR tablet (T20) reached Tmax at 4 h with Cmax 2400 ng/mL, while the floating SR design with an air compartment (T21) delayed Tmax to 5.5 h and reduced Cmax to 1850 ng/mL. Both maintained extended plasma levels over 24 h, reflecting controlled release and gradual elimination. Regional absorption shifted toward the jejunum and ileum, supporting the SR mechanism. The GastroPlus^®^ simulations provide a robust basis for establishing in vitro–in vivo correlation and predict that formulation design may significantly influence both absorption kinetics and systemic drug exposure [30].

## 4. Conclusions

This study successfully demonstrated the integration of HME and FDM 3DP for the fabrication of ketoprofen-loaded IR and SR oral tablets. Optimized filaments based on Kollicoat^®^ IR and HPMC exhibited suitable mechanical, thermal, and physicochemical properties, enabling robust printability. The resulting tablets met pharmacopeial quality standards, with dissolution profiles closely matching market ketoprofen formulations. IR tablets achieved rapid drug release (>80% in 30 min), while SR tablets extended drug release for up to 12 h. Release kinetics were primarily governed by infill density and shell number, with infill density exerting the stronger influence. In silico GastroPlus^®^ simulations further supported their translational potential by predicting pharmacokinetic parameters consistent with clinical data.

Compared to earlier studies in the field, which typically addressed either IR or SR profiles in isolation, this work provides a more comprehensive workflow encompassing filament engineering, advanced solid-state characterization, pharmacopeial quality control, comparative dissolution, and pharmacokinetic modeling. This breadth strengthens the translational relevance and highlights the potential of 3D printing for personalized oral drug delivery.

The study demonstrates the feasibility of integrating hot-melt extrusion and FDM 3D printing to produce both IR and SR ketoprofen tablets within a single workflow. The optimized filaments (F6, F13) provided robust printability and reproducible mechanical and dissolution performance.

Our IR tablets (T4, T5) matched market Profenid^®^ in dissolution and disintegration profiles, while SR tablets (T20, T21) closely mimicked Bi-Profenid^®^. These findings confirm that digital design parameters (infill density, shell number, air compartments) translate directly into functional release behaviors, as also observed by recent reports [refs 2021–2024]. In particular, using Micro-CT to validate internal architecture provides novel insight into structure–performance relationships, which has been underexplored in earlier studies.

This study demonstrated a unified FDM 3D printing workflow for fabricating IR and SR ketoprofen tablets from optimized HME filaments. The results establish a robust link between formulation, geometry, and drug release, validated through pharmacopeial quality testing, comparative dissolution with market products, and in silico GastroPlus^®^ simulations.

However, several limitations must be acknowledged. First, only ketoprofen was evaluated as a model drug. While informative, this restricts generalizability, since drugs with different solubility, thermal stability, or dose requirements may not behave similarly under identical processing conditions. Second, no in vivo studies were performed; although in silico predictions suggest translational potential, animal or human pharmacokinetic validation is essential before regulatory consideration. Third, the long-term stability of the filaments and printed tablets under various storage conditions was not assessed, leaving open questions regarding amorphous drug recrystallization and shelf-life. Finally, scalability remains uncertain, as manual filament feeding and bench-scale extrusion may not reflect industrial practice.

While FDM 3D printing is well-suited for thermally stable drugs such as ketoprofen, other additive manufacturing approaches offer solutions for thermolabile APIs. Techniques such as semi-solid extrusion (SSE), inkjet printing, and binder jetting enable fabrication under ambient or low-temperature conditions, minimizing heat exposure during processing. Additionally, hybrid strategies combining hot-melt extrusion for filament production with subsequent solvent deposition or post-loading steps can expand applicability to heat-sensitive compounds. Such approaches may complement FDM and allow future exploration of a broader range of active pharmaceutical ingredients.

By explicitly addressing these gaps, future research should explore broader drug classes, conduct stability and in vivo validation studies, and investigate scalable, GMP-compliant workflows. Despite these limitations, the findings provide a strong foundation for advancing 3D-printed personalized medicines toward clinical and regulatory acceptance.

Future applications of this platform extend beyond proof-of-concept. By leveraging FDM’s digital adaptability, such systems could enable point-of-care manufacturing in hospital pharmacies, telepharmacy-based dose personalization, and rapid prototyping of patient-specific dosage forms. Additionally, incorporating multi-drug polypills or layered release systems is feasible with the same workflow, providing opportunities for chronic disease management where flexible dosing and adherence are critical. Regulatory acceptance will depend on demonstrating filament stability, scale-up feasibility, and in vivo confirmation, but our findings provide a strong translational foundation for clinical integration.

## Figures and Tables

**Table 1:** Composition and processing parameters of drug-loaded filaments fabricated via hot-melt extrusion using different polymers and additives.

Filament Formulation	Polymer Type	Drug Loading (% *w*/*w*)	Extrusion Temperature (°C)	Screw Speed (rpm)	Additives
F1	Kollicoat^®^ IR	10%	160	30	
F2	Kollicoat^®^ IR	30%	160	30	
F3	Kollicoat^®^ IR	50%	160	30	
F4	Kollicoat^®^ IR	30%	180	30	
F5	Kollicoat^®^ IR	30%	160	50	
F6	Kollicoat^®^ IR	30%	160	30	1% Sorbitol + 1% SLS
F7	Kollicoat^®^ IR	30%	160	30	1% Sorbitol + 1% Tween
F8	Kollicoat^®^ IR	30%	160	30	1% Sorbitol
F9	PVA	30%	160	30	1% Sorbitol
F10	Kollidon^®^ SR	30%	160	30	1% Sorbitol
F11	Ethyl cellulose	30%	160	30	1% Sorbitol
F12	HPMC 2600–5600 cP	30%	160	30	
F13	HPMC 2600–5600 cP	30%	160	30	1% Sorbitol
F14	HPMC 2600–5600 cP	30%	160	30	1% Sorbitol + 1%SLS
F15	HPMC 2600–5600 cP	30%	160	30	1% Sorbitol + 1% Tween

**Table 2:** Mean weight, diameter (mm), and actual drug content (%, *w*/*w*) of extruded filaments. Data are presented as mean ± SD (*n* = 10).

Filament	Mean Weight (g) ± SD	Mean Diameter (mm) ± SD	Mean Actual Drug Content (%) ± SD
F1	0.097 ± 0.0032	1.704 ± 0.012	95.14 ± 2.15
F2	0.1032 ± 0.0028	1.702 ± 0.014	94.15 ± 2.05
F3	0.102 ± 0.0041	1.707 ± 0.015	82.47 ± 3.62
F4	0.103 ± 0.0030	1.698 ± 0.018	90.12 ± 2.48
F5	0.102 ± 0.0033	1.714 ± 0.016	92.14 ± 2.71
F6	0.101 ± 0.0035	1.707 ± 0.011	100.92 ± 1.93
F7	0.1056 ± 0.0042	1.7077 ± 0.014	93.24 ± 2.37
F8	0.10397 ± 0.0036	1.7011 ± 0.012	102.11 ± 2.15
F9	0.109 ± 0.0034	1.703 ± 0.013	99.60 ± 2.12
F10	0.142 ± 0.0040	1.819 ± 0.021	89.95 ± 2.68
F11	0.1236 ± 0.0037	1.550 ± 0.019	77.11 ± 4.01
F12	0.1622 ± 0.0035	1.735 ± 0.016	97.52 ± 2.10
F13	0.1625 ± 0.0034	1.732 ± 0.015	99.32 ± 1.92
F14	0.1622 ± 0.0033	1.732 ± 0.017	96.31 ± 2.25
F15	0.1623 ± 0.0031	1.734 ± 0.013	99.19 ± 1.89

**Table 3:** Mechanical characterization of extruded filaments based on mean break force, break stress, break stroke, break strain, break time, and maximum force. Data presented as mean ± standard deviation (*n* = 3).

Formulation	Mean Break Force (N)	Mean Break Stress (N/mm^2^)	Mean Break Stroke (mm)	Mean Break Strain (%)	Mean Break Time (s)	Mean Max Force (N)
F1	37.11 ± 1.43	47.25 ± 1.88	5.10 ± 0.44	5.10 ± 0.44	20.38 ± 1.15	50.06 ± 1.77
F2	31.35 ± 0.44	41.18 ± 0.63	7.91 ± 0.39	7.91 ± 0.39	19.62 ± 1.04	39.50 ± 0.53
F3	3.85 ± 0.28	2.33 ± 0.21	0.94 ± 0.08	1.14 ± 0.09	11.32 ± 0.91	4.43 ± 0.22
F4	26.68 ± 0.19	33.98 ± 0.74	49.80 ± 0.34	49.80± 0.34	34.14± 0.24	34.40 ± 0.84
F5	3.88 ± 0.29	4.94 ± 0.32	5.20 ± 0.42	5.20 ± 0.42	26.79 ± 1.10	4.24 ± 0.27
F6	79.65 ± 2.14	100.15 ± 3.22	3.89 ± 0.36	3.89 ± 0.36	15.56 ± 0.98	84.04 ± 2.11
F7	25.66 ± 1.13	8.17 ± 0.74	33.55 ± 2.61	67.09 ± 3.88	402.56 ± 6.21	45.82 ± 1.88
F8	56.15 ± 1.76	71.49 ± 2.10	6.37 ± 0.50	6.37 ± 0.50	25.49 ± 1.16	81.69 ± 2.35
F9	31.87 ± 1.24	40.57 ± 1.76	0.81 ± 0.07	0.81 ± 0.07	3.23 ± 0.20	32.17 ± 1.30
F10	31.27 ± 0.91	40.35 ± 1.02	71.58 ± 1.40	71.58 ± 1.40	42.67 ± 2.32	39.27 ± 0.97
F11	10.35 ± 0.40	13.18 ± 0.56	4.91 ± 0.35	4.91 ± 0.35	19.62 ± 0.92	10.50 ± 0.48
F12	61.88 ± 0.52	80.40 ± 0.71	3.73 ± 0.48	3.73 ± 0.48	20.92 ± 1.15	3.59 ± 0.80
F13	82.83 ± 3.24	108.01 ± 3.11	5.93 ± 0.75	5.93 ± 0.75	22.35 ± 0.92	102.77 ± 2.98
F14	80.83 ± 2.34	108.01 ± 3.11	6.37 ± 0.52	6.37 ± 0.52	25.71 ± 1.22	82.77 ± 2.98
F15	14.19 ± 0.38	4.97 ± 0.61	0.70 ± 0.63	0.70 ± 0.63	6.17 ± 2.04	14.85 ± 0.52
PVA (MakerBot)	41.74 ± 1.75	53.15 ± 2.13	1.43 ± 0.12	1.43 ± 0.12	5.71 ± 0.37	41.74 ± 1.75

**Table 4:** Design dimensions and average printing time for 3DP tablets with varying drug doses and release profiles. Data are presented as body dimensions (x × y × z in mm) and mean time ± SD (min) required to print each tablet design.

3DP Tablet	Body Design Dimension x × y × z mm	Mean Time to Print Body/min
Immediate 50 mg	7 × 7 × 4	2.75 ± 0.10
Immediate 75 mg	9 × 9 × 4	3 ± 0.12
Immediate 100 mg	9.25 × 9.25 × 5	3.75 ± 0.20
Sustained	10.5 × 10.5 × 5	5.5 ± 0.15
Sustained Air	11 × 11 × 5	6 ± 0.25

**Table 5:** The printing parameters and formulation characteristics of 3DP tablets include design type, printing pattern, shell number, infill density, printing speed, extrusion temperature, dimensional accuracy, drug amount per tablet, and polymer type used.

Formulation	Special Design	Printing Pattern	Shell Number	Infill Density %	Printing Speed Mm/s	Temp °C	Accuracy /mm	Drug Amount /mg	Polymer
T1	No air pocket	One wall	1	1	30	180	0.2	100	Kollicoat^®^ IR
T2	No air pocket	One wall	5	1	30	180	0.2	100	Kollicoat^®^ IR
T3	No air pocket	One wall	10	1	30	180	0.2	100	Kollicoat^®^ IR
T4	No air pocket	One wall	1	25	30	180	0.2	100	Kollicoat^®^ IR
T5	No air pocket	One wall	5	25	30	180	0.2	100	Kollicoat^®^ IR
T6	No air pocket	One wall	10	25	30	180	0.2	100	Kollicoat^®^ IR
T7	No air pocket	One wall	1	50	30	180	0.2	100	Kollicoat^®^ IR
T8	No air pocket	One wall	5	50	30	180	0.2	100	Kollicoat^®^ IR
T9	No air pocket	One wall	10	50	30	180	0.2	100	Kollicoat^®^ IR
T10	No air pocket	Tri hexa	5	25	30	180	0.2	100	Kollicoat^®^ IR
T11	No air pocket	Grid	5	25	30	180	0.2	100	Kollicoat^®^ IR
T12	No air pocket	One wall	5	25	30	200	0.2	100	Kollicoat^®^ IR
T13	No air pocket	One wall	5	25	30	180	0.2	50	Kollicoat^®^ IR
T14	No air pocket	One wall	5	25	30	180	0.2	75	Kollicoat^®^ IR
T15	No air pocket	Grid	10	50	30	180	0.2	150	HPMC 2600–5600 cP
T16	No air pocket	Grid	50	50	30	180	0.2	150	HPMC 2600–5600 cP
T17	No air pocket	Grid	100	50	30	180	0.2	150	HPMC 2600–5600 cP
T18	No air pocket	Grid	10	100	30	180	0.2	150	HPMC 2600–5600 cP
T19	No air pocket	Grid	50	100	30	180	0.2	150	HPMC 2600–5600 cP
T20	No air pocket	Grid	100	100	30	180	0.2	150	HPMC 2600–5600 cP
T21	Air pocket	Grid	100	100	30	180	0.2	150	HPMC 2600–5600 cP

**Table 6:** Physical characterization of 3D-Printed ketoprofen tablets (T5, T13, T14, T20, and T21). Results represented as mean ± SD.

Parameter	T5 (IR 100 mg)	T13 (IR 50 mg)	T14 (IR 75 mg)	T20 (SR 150 mg)	T21 (SR Air 150 mg)
Weight (g) Mean ± SD	0.350 ± 0.006	0.170 ± 0.004	0.260 ± 0.005	0.520 ± 0.007	0.537 ± 0.008
Diameter (mm) Mean ± SD	9.23 ± 0.06	7.02 ± 0.04	9.03 ± 0.07	10.52 ± 0.08	11.03 ± 0.06
Thickness (mm) Mean ± SD	4.96 ± 0.03	4.04 ± 0.02	3.96 ± 0.04	5.02 ± 0.04	5.09 ± 0.03
Hardness (kg) Mean ± SD	4.49 ± 0.14	4.42 ± 0.12	4.56 ± 0.13	7.34 ± 0.16	7.39 ± 0.15
Friability (%) Mean ± SD	0.34 ± 0.03	0.28 ± 0.02	0.30 ± 0.03	0.21 ± 0.02	0.23 ± 0.01
Disintegration Time (min) Mean ± SD	28.46 ± 0.71	20.13 ± 0.68	22.47 ± 0.74	N/A	N/A
Content Uniformity (%)Mean ± SD)	99.5 ± 1.3	99.7 ± 1.17	100.75 ± 1.71	99.1 ± 1.6	98.0 ± 1.8
Assay (%) Mean ± SD)	99.5 ± 1.1	99.7 ± 1.3	100.8 ± 1.2	100.2 ± 1.5	98.0 ± 1.7

**Table 7:** Comparison of dissolution profiles for formulations T4 and T5 with reference Profenid^®^ 100 mg using model-independent parameters.

Formulation	f1 (Difference Factor)	f2 (Similarity Factor)
T4	1.59	78.4
T5	2.87	74.6

**Table 8:** Comparison of dissolution profiles for formulations T20 and T21 with reference Bi-Profenid^®^ 150 mg using model-independent parameters.

Formulation	f1 (Difference Factor)	f2 (Similarity Factor)
T20	8.93	62.4
T21	12.6	56.2

**Table 9:** Pharmacokinetic parameters from GastroPlus^®^ simulations.

Formulation	Dose (mg)	Tmax (h)	Cmax (ng/mL)	AUC (ng.h/mL)
T13	50	1.1	2600	5460
T14	75	1.1	3800	9900
T5	100	1.1	5400	13,200
T20	150	4.0	2400	22,380
T21	150	5.5	1850	21,610

## Data Availability

The original contributions presented in this study are included in the article. Further inquiries can be directed to the corresponding authors.

## Abbreviations

The following abbreviations are used in this manuscript:

3DP	Three-Dimensional Printing
AM	Additive Manufacturing
API	Active Pharmaceutical Ingredient
ATR-FTIR	Attenuated Total Reflection Fourier Transform Infrared
BV	Bone Volume
DSC	Differential Scanning Calorimetry
FDM	Fused Deposition Modeling
HCl	Hydrochloric Acid
HME	Hot-Melt Extrusion
HPMC	Hydroxypropyl Methylcellulose
HPC	Hydroxypropyl Cellulose
IR	Immediate Release
IVIVC	In Vitro–In Vivo Correlation
KIR	Kollicoat^®^ IR
NSAID	Non-Steroidal Anti-Inflammatory Drug
PVA	Polyvinyl Alcohol
PVP	Polyvinylpyrrolidone
SD	Standard Deviation
SEM	Scanning Electron Microscopy
SLS	Sodium Lauryl Sulfate
SR	Sustained Release
TGA	Thermogravimetric Analysis
Tmax	Time to Maximum Plasma Concentration
Cmax	Maximum Plasma Concentration
AUC	Area Under the Curve
USP	United States Pharmacopeia
XRD	X-ray Diffraction
